# *Cbx2*, a PcG Family Gene, Plays a Regulatory Role in Medaka Gonadal Development

**DOI:** 10.3390/ijms21041288

**Published:** 2020-02-14

**Authors:** Qinghe Chao, Fengfeng Shen, Yidong Xue, Jikui Wu, Junling Zhang

**Affiliations:** 1 Key Laboratory of Freshwater Aquatic Genetic Resources, Ministry of Agriculture, Shanghai Collaborative Innovation for Aquatic Animal Genetics and Breeding, National Demonstration Center for Experimental Fisheries Science Education, Shanghai Ocean University, Shanghai 201306, China; 19821252732@163.com (Q.C.); 18336072982@163.com (F.S.); 13916157525@163.com (Y.X.); 2 Laboratory for Marine Fisheries Science and Food Production Processes, Qingdao National Laboratory for Marine Science and Technology, Qingdao 266071, China; 3 Laboratory of Quality and Safety Risk Assessment for Aquatic Product on Storage and Preservation, Ministry of Agriculture, Shanghai Ocean University, Shanghai 201306, China

**Keywords:** *cbx2*, gonadal development, medaka

## Abstract

Chromobox homolog 2 (CBX2), a key member of the polycomb group (PcG) family, is essential for gonadal development in mammals. A functional deficiency or genetic mutation in *cbx2* can lead to sex reversal in mice and humans. However, little is known about the function of *cbx2* in gonadal development in fish. In this study, the *cbx2* gene was identified in medaka, which is a model species for the study of gonadal development in fish. Transcription of *cbx2* was abundant in the gonads, with testicular levels relatively higher than ovarian levels. In situ hybridization (ISH) revealed that *cbx2* mRNA was predominately localized in spermatogonia and spermatocytes, and was also observed in oocytes at stages I, II, and III. Furthermore, *cbx2* and *vasa* (a marker gene) were co-localized in germ cells by fluorescent in situ hybridization (FISH). After *cbx2* knockdown in the gonads by RNA interference (RNAi), the sex-related genes, including *sox9* and *foxl2,* were influenced. These results suggest that *cbx2* not only plays a positive role in spermatogenesis and oogenesis but is also involved in gonadal differentiation through regulating the expression levels of sex-related genes in fish.

## 1. Introduction

The polycomb group (PcG) protein family was initially identified in *Drosophila* [1]. In higher eukaryotes, it is mainly involved in maintaining a series of cellular physiological activities, such as cell differentiation, cell cycle regulation, cell senescence, and X chromosome inactivation [2]. PcG proteins are assembled into two Polycomb Repressive Complexes, PRC1 and PRC2 [3]. In embryonic stem cells, whole-genome target analysis revealed that the two complexes were localized in the promoter region of developmental regulators [4]. Functionally, PRC1 and PRC2 exhibit enzymatic activities to modify histones and are crucial for epigenetic regulation via histone methylation [5,6,7].

As a core component of PRC1, chromobox (CBX) homolog protein performs an indispensable function regarding stem cell maintenance and embryo development [8]. In mammals, the CBX family contains five proteins, CBX2, CBX4, CBX6, CBX7, and CBX8 [9]. Although they have a common conserved domain, their molecular size, tissue distribution, and biological functions are quite different. In recent years, *cbx2* has become increasingly important due to the discovery of the role of CBX2 in human and mouse gonadal development. In mice, a lack of *cbx2* can lead to gonadal hypoplasia and sex reversal (testis–ovary) [7]. Sex reversal was also observed in humans due to *cbx2* gene mutation [10]. The CBX2 protein mainly participates in the recruitment and stabilization of PRC1 in mitotic chromosomes [11]. Previous reports suggested that CBX2, a member of PcG, was an epigenetically modified transcriptional repressor. Recently, it was also found to serve as a transcriptional activator to regulate the expression of downstream genes [12,13]. However, the functional role of *cbx2* in gonadal development is still unclear in vertebrates other than mammals.

Medaka (*Oryzias latipes*) is regarded as a model animal to investigate the gonadal development of vertebrates [14]. Like mammals, medaka has XY sex chromosomes and was the first species to define the sex-determining gene *dmy* in fish [15,16]. Due to its small size (2-3 cm), short sexual maturity cycle (3 months), and gender plasticity, medaka is usually used to explore gonadal development in vertebrates. In this work, we identified medaka *cbx2* via PCR cloning and sequencing, detected the relative expression of *cbx2* mRNA in different tissues using real-time PCR, and established an expression profile of *cbx2* mRNA in medaka gonads via digoxigenin (DIG)-labeled in situ hybridization (ISH) and fluorescent in situ hybridization (FISH). Furthermore, the expression of *cbx2* mRNA was knocked down using RNA interference (RNAi), then the expression levels of *cbx2* and sex-related genes, including *foxl2* and *sox9,* were analyzed to clarify the function of *cbx2* in the gonadal development of fish.

## 2. Results

### 2.1. Molecular Characterization and Phylogenetic Analyses of cbx2

**Figure 1:**
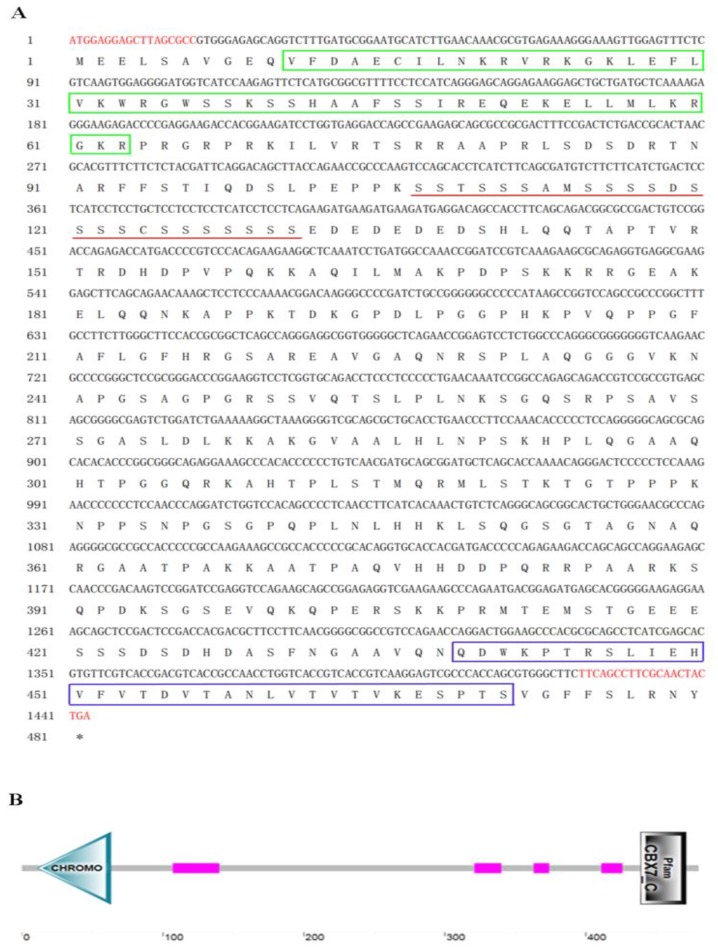
The cDNA sequences and the derived protein sequence of medaka *cbx2* (**A**). The red font position represents the cloning primers, the Chromo domain is marked by a green box, the Pfam domain is marked by a blue box, and the underlined sequence indicates the serine-rich region. The secondary structure of CBX2 in medaka (**B**).

**Figure 2:**
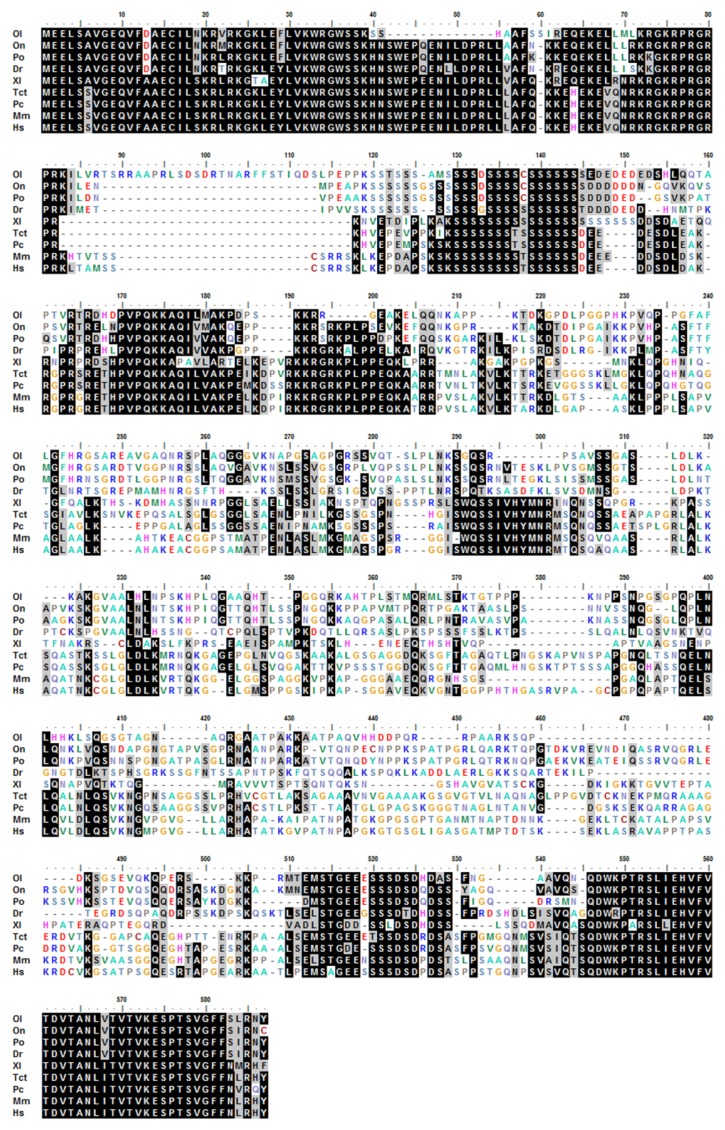
Sequence alignment of the CBX2 protein between different species. Abbreviations: Ol, *Oryzias latipes*; On, *Oreochromis niloticus*; Po, *Paralichthys olivaceus*; Dr, *Danio rerio*; Xl, *Xenopus laevis*; Tct, *Terrapene carolina triunguis*; Pc, *Phasianus colchicus*; Mm, *Mus musculus*; Hs, *Homo sapiens*.

**Figure 3:**
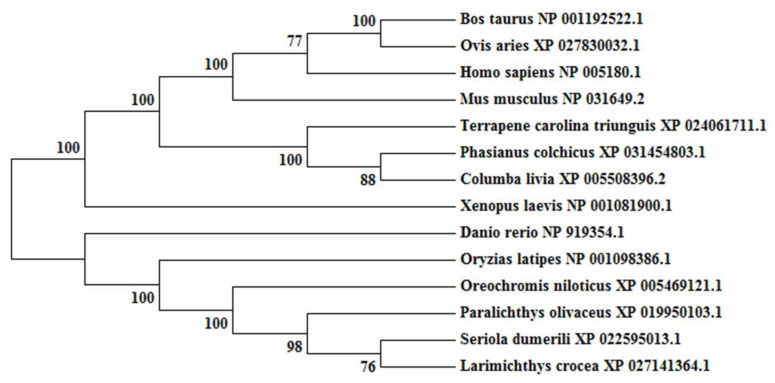
Phylogenetic tree of CBX2 in vertebrates. Bootstrap values are marked at the branch points.

The obtained cDNA of cbx2 was 1443 bp in length and encoded 480 amino acid residues (Figure 1A). The molecular mass of the deuced CBX2 protein was 51.5 kDa and its theoretical isoelectric point was 10.12. Among these amino acids, the ratios of serine were the highest (14.0%). The CBX2 protein had no signal peptide or transmembrane region, indicating that it was neither secreted nor transmembrane-located. It contained one Chromo domain (11–63 bp), four low complex domains, and a Pfam domain (439–471 bp) (Figure 1B). Comparing the amino acid sequences of CBX2 in different species, we found that the anterior and posterior amino acid sequences of the CBX2 protein were more conservative and the middle parts were less conservative (Figure 2). A phylogenetic tree showed that CBX2 was evolutionarily conserved from fish to humans. The CBX2 proteins of medaka and all other fish were grouped together, whereas those of amphibians, reptiles, birds, and mammals were grouped together (Figure 3).

### 2.2. Tissue Expression of cbx2 mRNA

**Figure 4:**
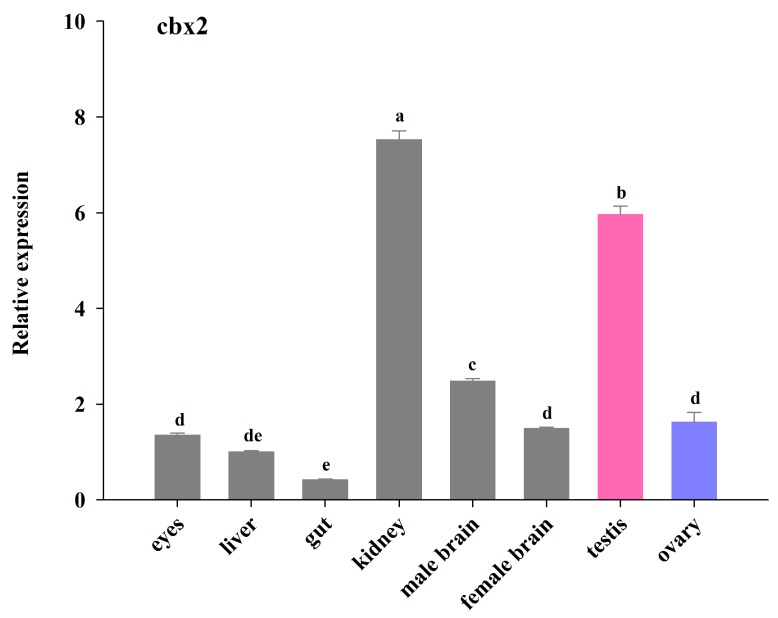
The relative expression of *cbx2* mRNA in different medaka tissues. The different alphabetic signs indicate statistically significant differences (*P* < 0.05).

The relative expression of *cbx2* mRNA in different tissues was evaluated using real-time PCR (Figure 4). The *cbx2* mRNA was detected in different tissues of the adult medaka. The expression of *cbx2* mRNA was higher in the kidneys and testes than in other tissues. Furthermore, the expression of *cbx2* mRNA in the testes was about three times that in the ovaries, and its level in male brains was relatively higher than in female brains (*P* < 0.05).

### 2.3. Gonadal Localization of cbx2 mRNA by ISH and FISH

**Figure 5:**
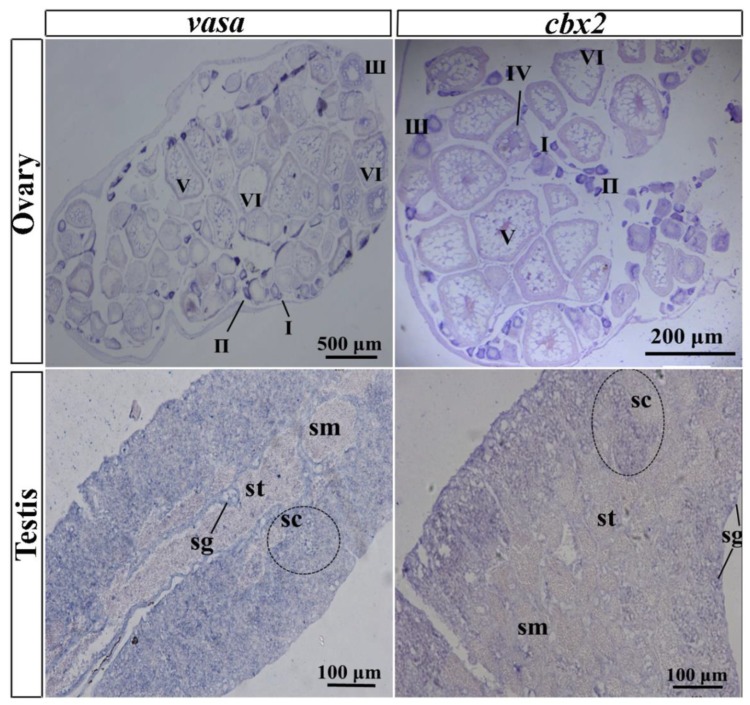
Digoxigenin (DIG)-labeled in situ hybridization (ISH) in medaka ovaries and testes. Paraffin sections were hybridized with the antisense probe of *cbx2* and *vasa*. Signals were visualized using chromogenic staining. I–VI, oocyte stage; sg, spermatogonia; sc, spermatocytes; st, spermatid; sm, sperm.

In the ovary, oocyte development is not synchronized because adult female medaka produce eggs daily. A small number of oocytes exist at stage I and a large number of oocytes exist in the ovary from stage II to stage V. In this study, *vasa* was employed as a marker gene, with its expression pattern first being verified by DIG-labeled ISH. The results showed that *vasa* mRNA was expressed throughout the whole oogenesis process, with higher expression in stages I, II, and III, whereas it was expressed at a lesser extent in stages IV and V. Similarly, *cbx2* mRNA was highly expressed in oocytes at stages I, II, and III, and expressed less in stage IV and V oocytes (Figure 5). No positive signals were sensed by ISH in the probe.

Medaka testes are small in size, and the reproductive epithelium divides them into many irregular efferent cysts containing a variety of germ cells during gametogenesis (Figure 5). From the outside in, a testis is divided into spermatogonia, spermatocytes, spermatids, and sperm. The ISH results showed that *cbx2* mRNA was chiefly located in spermatocytes and spermatogonia, and it was hardly distributed at all in mature sperms and spermatids (Figure 5). Similarly, *vasa* mRNA was localized predominately in the spermatogonia and spermatocytes, with no obvious signal in sperm. 

**Figure 6:**
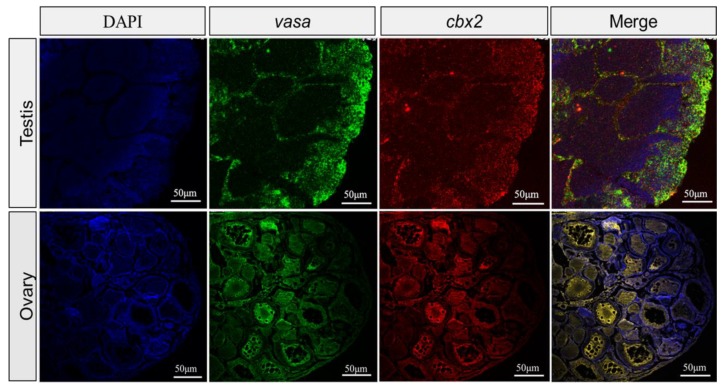
Fluorescent in situ hybridization (FISH) in medaka ovaries and testes. The mRNA signals were stained with fluorescence, with green indicating *vasa* and red indicating *cbx2,* and the nuclei were dyed with DAPI (blue).

To further localize the *cbx2* mRNA, we utilized *vasa* as a reference to perform FISH. The results showed that the cellular localization of *cbx2* and *vasa* mRNA almost completely overlapped in the ovary and the testis (Figure 6), which was consistent with the ISH results.

### 2.4. Expression Changes of cbx2, sox9, and foxl2 mRNA after RNA Interference

**Figure 7:**
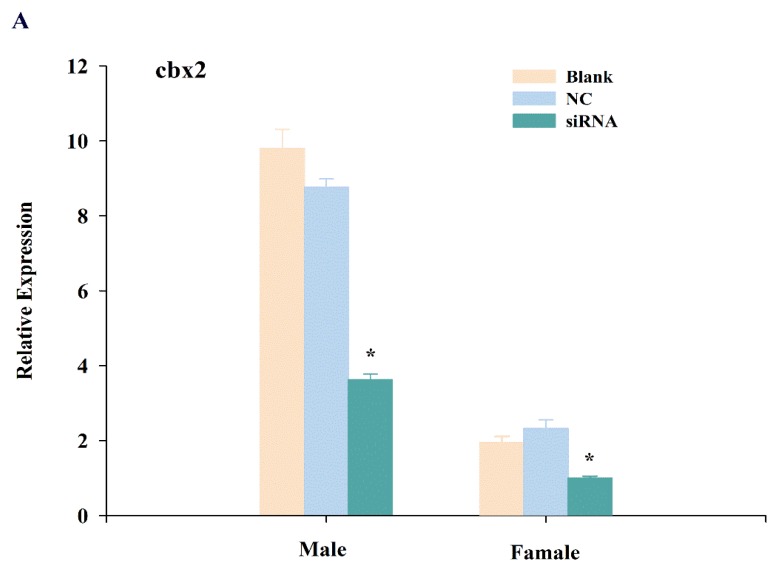

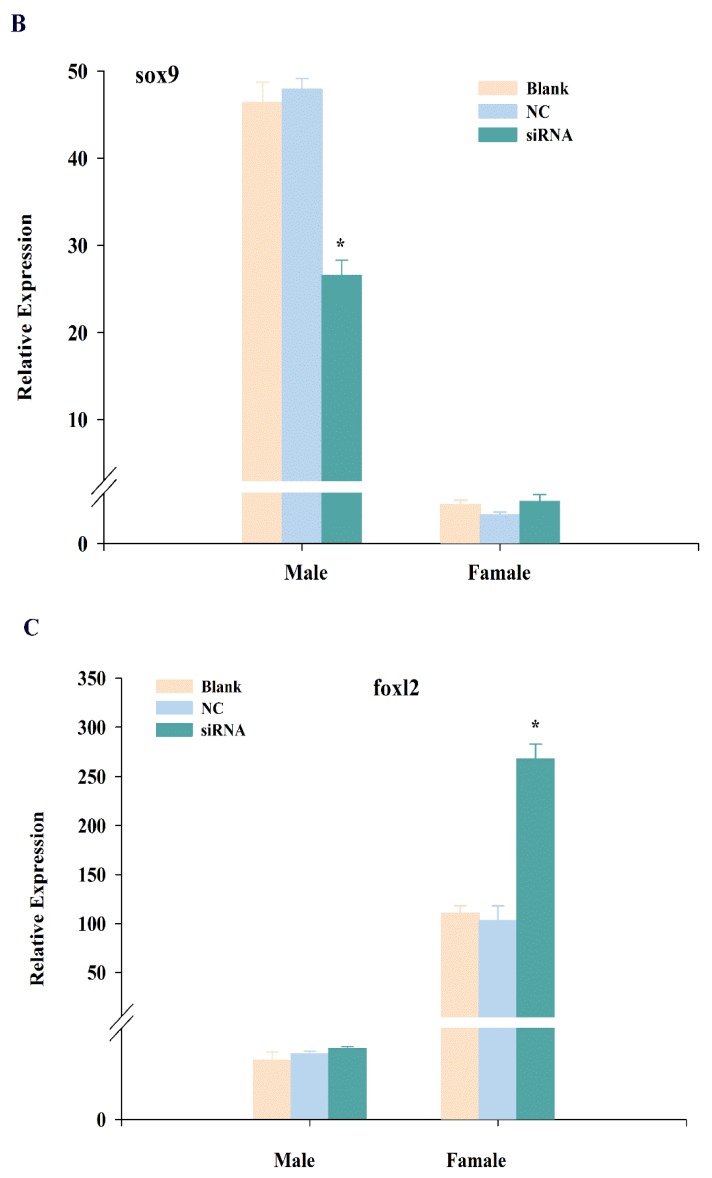
Expression changes of *cbx2* (**A**), *sox9* (**B**), and *foxl2* (**C**) mRNA in gonads after RNA interference. The asterisk represents a statistically significant difference (*P* < 0.05).

To further explore the function of *cbx2*, we knocked down *cbx2* and investigated the expression changes of sex-related genes (i.e., *sox9* and *foxl2*). The results showed that the expression of *cbx2* in the testis was distinctly inhibited after injecting siRNA (*P* < 0.05), and its expression was also significantly decreased in the ovary (Figure 7A). Furthermore, the level of *sox9*, a male sex-related gene, was decreased in the testis when *cbx2* expression was inhibited (Figure 7B). However, the level of *foxl2,* a female sex-related gene, was increased in the ovary (Figure 7C).

## 3. Discussion

In this study, *cbx2* was identified in medaka and shown to be conserved in vertebrate evolution. The CBX2 protein had the highest ratio of serine. Kawaguchi et al. found that the serine-rich region is the phosphorylation site of CBX2 and is stably phosphorylated in cells [17]. In fish-to-human evolution, the length of the serine-rich region decreased from 24 serine residues to 16 serine residues [18]. Sequence alignment suggested that the anterior-Chromo domain and the posterior-Pfam domain possessed high sequence similarity. H3K27me3 is a typical epigenetic gene silencing marker, often located in the promoter region of developmental genes. The Chromo domain recognizes H3K27me3 and recruits PRC1 to the target gene to regulate gene expression. The Pfam domain is a conserved region at the C-terminus of the CBX protein family and is involved not only in transcriptional silence but also often binds the RING1B domain, causing the protein complexes to participate in the alternative recruitment pathway of chromatin targeting [19]. These results suggest that the CBX2 protein plays an important role in epigenetic regulation.

This work showed that *cbx2* was expressed relatively abundantly in medaka gonads and kidneys. In humans, RNA sequencing data analysis revealed that *cbx2* was more abundant in testes, followed by ovaries, placenta, and then lungs, but was expresses to a lesser extent in other tissues [20]. In mice, sequencing results showed that *cbx2* was expressed mainly in the brain and limbs, with greater expression also gonads but less in other tissues. Moreover, our real-time PCR results indicated that the expression of *cbx2* in testes was higher than in ovaries, suggesting that *cbx2* may perform a vital function regarding gonadal development. In addition, *cbx2* is also involved in balancing the self-renewal and differentiation of haematopoietic stem cells [21], lymphopoiesis [22], and the antiviral immunity response [23]. The high expression of *cbx2* in medaka kidneys indicates that *cbx2* is associated with the immune system. 

Furthermore, our results from ISH and FISH showed that the expression profile of *cbx2* was similar to that of *vasa* in medaka gonads. *Vasa* is an evolutionarily conserved translation factor and is required for the polarization of the anterior–posterior axis and the formation of germ cells. So far, the expression of *vasa* mRNA has only been found in medaka germ cells [24]. Similar to *vasa*, *cbx2* signals were mostly localized in spermatocytes and spermatogonia and not so much in mature sperms and spermatids in medaka testes. In medaka ovaries, *cbx2* was also found in primary oocytes at stages I, II, and III. Previous studies in mammals showed that *cbx2* plays a key role in cell cycle changes [25,26], meiosis, homologous chromosome synapsis, and germ cell differentiation [27]. These results suggest that *cbx2* might be involved in spermatogenesis and oogenesis in medaka.

After *cbx2* knockdown in medaka, *sox9* expression was downregulated in testes. SRY-related HMG box 9 (*sox9)* is related to spermatogenesis and testis maintenance and development in mammals. Johnsen et al. found that *sox9* negatively regulated the expression of aromatase to inhibit estrogen synthesis [28], with aromatase being the rate-limiting enzyme in estrogen synthesis [29]. Therefore, the high expression of *sox9* may be one of the key factors in gonad masculinization. In teleost, *sox9* is also significantly expressed during gonadal development and affects the differentiation process of both germ cells and supporting cells [30]. As a result, we speculated that *cbx2* was possibly involved in male gonadal development by regulating *sox9* expression in medaka.

On the other hand, the expression of sex-related *foxl2* was upregulated in ovaries when *cbx2* expression was inhibited in medaka. Forkhead transcriptional factor 2 (*foxl2)* is important in ovarian development and a key factor in female sex determination, and was also found to be abundantly expressed in *Gobiocypris rarus* [31] and *Clarias gariepinus* [32] ovaries. *Foxl2* was shown to affect gonadal differentiation and development by regulating *cyp19a1*, a key gene in the female signaling pathway in *Oreochromis niloticus* [33]. These results indicate that *cbx2* is also related to female gonadal development by controlling the gene expression of *foxl2* in medaka.

In mice, *M33* (*cbx2*) deficiency may cause sex reversal, i.e., male-to-female [34]. In humans, the expression levels of *Nr5a1*, *sox9,* and *sry* decrease significantly when *cbx2* is knocked down using siRNA, and target identification by genome-wide sequencing suggested that *cbx2* influences gonadal development by promoting the male signaling pathway while simultaneously inhibiting the female signaling pathway [35]. Therefore, the higher expression of *cbx2* in gonads implied that this gene plays a role in spermatogenesis and oogenesis, and the gene knockdown results demonstrated its involvement in gonadal differentiation by controlling the sex-related genes, such as *sox9* and *foxl2,* in medaka. 

## 4. Materials and Methods 

### 4.1. Experimental Materials

Medaka were raised at the Experimental Center of Shanghai Ocean University. Adult fishes at 3 months of age (n = 3 pools, 10 specimens/pool) were collected. The female brains, male brains, ovaries, testes, livers, kidneys, guts, and eyes were dissected, swiftly frozen, and stored at −80 °C in a refrigerator for RNA extraction. In addition, the ovaries and testes were cut and fixed in 4% paraformaldehyde overnight, then stored into 70% ethanol to make sections for ISH and FISH. Animal experiments were carried out in strict accordance with the guidance of the committee for the Laboratory Animal Research of Shanghai Ocean University.

### 4.2. Molecular Cloning and Sequence Analysis

Total RNA of different tissues was extracted using TRizol (Invitrogen, Carlsbad, CA, USA). To eliminate genome contamination, cDNA was reversed transcribed using the PrimeScript™ RT reagent Kit with the gDNA Eraser (Takara Biotechnology, Dalian, China). By searching the NCBI database, we obtained the predicted CDS sequences encoding *the* medaka *cbx2* gene (Genebank accession No. EF537027.1). The CDS region of *cbx2* was verified by PCR cloning and sequencing. Then, the physicochemical properties of the predicted proteins were analyzed online using the ExPASy-ProtParam tool, and the secondary and tertiary structures were predicted using SMART and Swiss-model. Protein sequence alignment was executed by BioEdit, and the neighbor-joining (NJ) tree was established by MEGA 7.0 based on CBX2 protein sequences from various species according to BLASTP. 

### 4.3. Real-Time PCR of cbx2 in Different Tissues

Real-time PCR was performed with specific primers of *cbx2* and 18S, as a reference gene, following the same set of cDNA amplification. The reaction underwent 39 cycles of 10 s at 95 °C and 30 s at 60 °C in a 20 μL system using CFX96 Touch real-time (Bio-Rad, Hercules, CA, USA). The PCR primers are described in Table 1.

The relative expression for *cbx2* mRNA was evaluated using the 2^-ΔΔCt^ method and the results were presented according to the mean ± standard deviation (SD). Statistical analysis was performed with one-way of variance (ANOVA) using SPSS 24 software. Differences were considered to be statistically significant at *P* < 0.05.

### 4.4. Gonadal Expression of cbx2 by ISH and FISH

In order to understand the subcellular distributions of *cbx2*, ISH and FISH were performed on gonadal sections and verified by hematoxylin and eosin staining. ISH was performed as previously described [24]. Briefly, the 552 nt of *cbx2* cDNA and 794 nt of *vasa* cDNA were inserted into a pGEM-T vector, respectively. The recombinant plasmids were linearized with spel or sphl to transcribe DIG-labeled or FITC-labeled antisense or sense probes according to RNA Labeling kit instructions (Roche, Basel, Switzerland). The synthetic probe was incubated with DNaseI (Thermo Fisher, Carlsbad, CA, USA) and precipitated by LiCl at –80 °C. The ISH experiments of *cbx2* and *vasa* as the marker gene were performed and stained with BCIP/NBT. FISH was carried out with a TSA™ Plus Fluorescence Systems kit according to the instructions (NEL756, PerkinElmer, Waltham, MA, USA). The nucleus was dyed with DAPI and the slide was fixed with Anti-Fade Mounting Medium (Invitrogen, Carlsbad, CA, USA).

Photography and observation of ISH were performed using an upright microscope (Leica DM500, Heerbrugg, Switzerland) and FISH images were observed and photographed using a Leica SP8 FALCON with the confocal image system (LAS X).

### 4.5. RNA Interference

siRNA sequences were designed and synthesized by Shanghai GeneBio Co. Ltd (Table 2). According to the instructions, *cbx2*-siRNA-1, *cbx2*-siRNA-2, and *cbx2*-siRNA-3 were mixed and diluted with Rnase-free water. For RNA interference, a pre-experiment was performed to optimize the injection concentration of the siRNA mixture (25 ng, 50 ng, 75 ng, or 100 ng). The optimal 50 ng siRNA mixture was then injected from the cloacal aperture of the medaka using a medical syringe with a diameter of 0.38 µm. A negative control of siRNA was synthesized, injected, and a blank group was set up synchronously. A total of 10 adult fishes at 3 months of age were injected in each assay. Each assay was repeated in triplicate.

After 48 h, the injected fishes were collected and their gonadal tissues were dissected for RNA extraction. Real-time PCR was performed to detect the expression changes of *cbx2*, *sox9,* and *foxl2* mRNA after RNA interference. The experimental methods and procedures were consistent with those described above. 

## Figures and Tables

**Table 1:** Primers for real-time PCR and probe synthesis.

Primer	Primer Sequence (5′→3′)
*cbx2*-F	GCCCAAGTCCAGCACCTCA
*cbx2*-R	GCTCCTTCGCCTCACCTCT
*cbx2*-spel-F	GGACTAGTTGGTGGGCGACTCCTTGA
*cbx2*-sphl-R	CATGCATGCACCTGAACCCTTCCAAACAC
*vasa*-spel-F	GGACTAGTCCCGCTTGTTGAATTTGG
*vasa*-sphl-R	CATGCATGCTGTGCGAGTCGTTGGAGAA
*sox9*-F	AAACTGGCCGACCAATAC
*sox9*-R	CTCAGCCTCCTCCACAAA
*foxl2*-F	TCCTACACGTCCTGCCAGAT
*foxl2*-R	CCCATGCCGTTGTAAGAGTT
*18s*-F	CTGAGAAACGGCTACCACAG
*18s*-R	CAGCAACTTTAAGATACGC

Codes were used to degenerate the primers. F, sense primer; R, antisense primer.

**Table 2:** siRNA target site and sequence.

Gene	Direction	Sequence
*cbx2*-siRNA-1(251)	Sense (5′-3′)	CCGACUCUGACCGCACUAATT
	Anti-sense (3′-5′)	UUAGUGCGGUCAGAGUCGGTT
*cbx2*-siRNA-2(853)	Sense (5′-3′)	GCGCUGCACCUGAACCCUUTT
	Anti-sense (3′-5′)	AAGGGUUCAGGUGCAGCGCTT
*cbx2*-siRNA-3(1337)	Sense (5′-3′)	GCCUCAUCGAGCACGUGUUTT
	Anti-sense (3′-5′)	AACACGUGCUCGAUGAGGCTT
